# Centrosome Positioning in *Dictyostelium*: Moving beyond Microtubule Tip Dynamics

**DOI:** 10.3390/cells7040029

**Published:** 2018-04-12

**Authors:** Michael P. Koonce, Irina Tikhonenko

**Affiliations:** Division of Translational Medicine, New York State Department of Health, Wadsworth Center, Albany, NY 12201-0509, USA; irina.tikhonenko@health.ny.gov

**Keywords:** centrosome, *Dictyostelium* sp., microtubule, dynein, kinesin

## Abstract

The variability in centrosome size, shape, and activity among different organisms provides an opportunity to understand both conserved and specialized actions of this intriguing organelle. Centrosomes in the model organism *Dictyostelium* sp. share some features with fungal systems and some with vertebrate cell lines and thus provide a particularly useful context to study their dynamics. We discuss two aspects, centrosome positioning in cells and their interactions with nuclei during division as a means to highlight evolutionary modifications to machinery that provide the most basic of cellular services.

## 1. Introduction

Centrosomes are versatile eukaryotic organelles that organize microtubule (MT) arrays during interphase and division in animal cells. All centrosomes must duplicate once per cell cycle and all play a role in MT nucleation. However, as likely highlighted in this volume, centrosome size, shape, and architecture can vary significantly in different organisms. Comparative analyses of centrosomes have fostered a better understanding of their core activities and have led to thoughtful examination of their origin and evolution [1,2,3,4]. Less understood are the functional components that make centrosomes different. For example, while over half of the *S. cerevisiae*, *S. pombe*, and human centrosome components are orthologous [5], the three organelles are significantly different in structure and cell cycle dynamics. The functional drivers of these differences are rooted in biological context and their study in multiple organisms can lend insight into the delicate interplay of machinery for both conserved and distinct activities. In this light, we focus on centrosomes in the social amoeba, *Dictyostelium discoideum*. This organism displays a hybrid range of centrosome activities, some seen in the well-studied fungal models and others in vertebrate cell lines. In our view, this offers an opportune window into how nature modifies machinery crucial for centrosome positioning and cell division.

**Figure 1:**
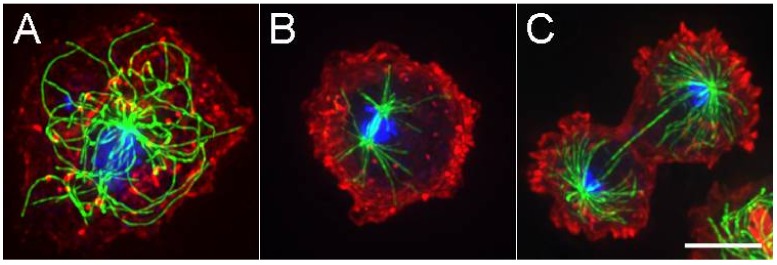
Microtubules (MTs) in *D. discoideum*. (**A**). Interphase cell with radially arranged MTs (in green) emanating from a centrally located centrosome. DNA is in blue, actin in red. Panels (**B**) and (**C**) show anaphase and late telophase views of mitotic spindle assemblies. While the spindle MTs are contained within a semi-closed nuclear compartment, astral MTs project off into the cytoplasm. Scale bar = 5 μm.

The *D. discoideum* centrosome consists of a multi layered, box-like core structure surrounded by an amorphous, electron dense corona [6,7]. The core is similar but significantly thicker than the layered structures found in the two well-studied fungi and is much different than the tubulin-based centrioles found in many animal cells. During interphase, the *D. discoideum* centrosome is located in the cytoplasm, separate from but firmly attached to the nucleus (hence it is also known as a nucleus-associated-body, or NAB). MTs originate from the corona and extend in a radial fashion typical of vertebrate tissue culture cells (Figure 1). The minus ends remain anchored in the corona and the distal segments frequently undergo rapid bending and lateral movements readily seen in live cell imaging.

On entry to mitosis, the cytoplasmic MT array dissolves, the centrosome begins to duplicate via longitudinal splitting of the core structure, inserts into an opening of the nuclear envelope, and organizes a compact intranuclear spindle similar in morphology to those seen in fungi (Figure 1) [7]. Pole separation during anaphase is extensive, facilitated by pulling forces acting on the spindle astral MTs and tempered by Kinesin-5 action in the spindle midzone [8,9]. As mitosis nears completion, astral MTs grow and the centrosome is extruded back into the cytoplasm to complete the cycle.

The differences in structure from either fungi or vertebrate cell centrosomes spark interesting questions regarding how *D. discoideum* processes events that should be broadly conserved (e.g., timing/regulation of mitotic entry, disassembly of interphase MTs) and of events specialized for *D. discoideum* biology (e.g., duplication of the centrosomal core, nuclear attachment). Though not unique, the lateral motions of the MTs during interphase and integration/extrusion of the centrosome into the nuclear envelope are exaggerated here and offer an opportunity to explore roles that motor proteins play during the centrosome cycle. To address these points further, we discuss how an interplay of motor activities influences MT movement and centrosome position and then what can be learned from uncoupling the interphase centrosome–nuclear connection.

## 2. *D. discoideum* Centrosome Position Is Driven by Motor Activity

Polymer assembly and disassembly reactions at the distal ends of MTs are well-known to create pushing and pulling forces that are minimally sufficient to self-center the centrosome. These movements can be recapitulated in defined artificial environments and understood via modeling [10,11]. This mechanism appears to be a dominant driver of centrosome positioning in many cell types. However, there are multiple other examples of centrosome movement to indicate there are additional components to its cellular positioning. Cortical pulling forces often engage astral MTs during cell division to displace the spindle and create asymmetric sized cells [12]; during interphase, centrosomes can be moved to the cell periphery where they act as basal bodies to nucleate axonemal structures [13,14]; centrosomes support nuclear migration during cell growth via coupling nuclei to distal forces acting on the cytoskeleton [15]. All these activities involve motors such as dynein and kinesin that push, pull, and depolymerize MTs, or engage actin filaments to effect centrosome position.

**Figure 2:**
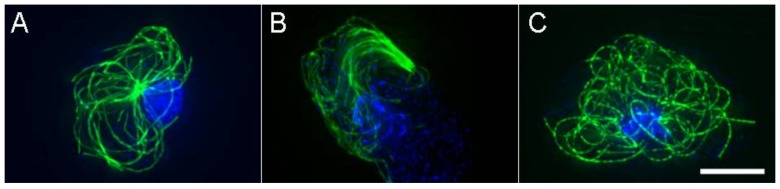
Variable interphase MT architecture. (**A**) Wild-type arrangement (**B**). Typical motile comet-like arrangement of MTs in cells with impaired dynein function. (**C**) Spaghetti-like MT pattern, in a cell where Kinesin-4 (Dd Kif8) function has been disrupted. Note here a loss of the distinctive radial MT pattern; even the centrosome position is non-distinct. MTs in green, Scale bar = 5 μm.

In *D. discoideum*, interphase MT ends do not appear to grow and shrink as much as they do in other more robust cell types and instead, the polymer shape and position is significantly influenced by cellular motor activity (Figure 2) [16,17]. For example, MTs readily bend back and forth in cytoplasm and arc along the cell cortex as if pushed and pulled by dynein or kinesin. Perturbations to the dynein motor or dynein accessory proteins result in a dramatic wholescale movement of the MT array [18,19]. In these cases, the centrosome leads a comet-like array of MTs through the cytoplasm, at rates up to 2.5 μm/s. Using a laser microbeam to sever MTs, we previously demonstrated that centrosome movement is at least partially effected through pushing forces acting on the trailing MTs [16].

Disruption of either Kinesin-8 or Kinesin-4 isoforms (Ddkif10, Ddkif8, respectively) was sufficient to abrogate the dynein perturbation and in part, perturb the radial character of the MT array [20]. Deletion of Kif8 or the MT associated protein, Ase1A was also sufficient to alter the radial character of the MT arrays (Figure 2) [9,20].

**Figure 3:**
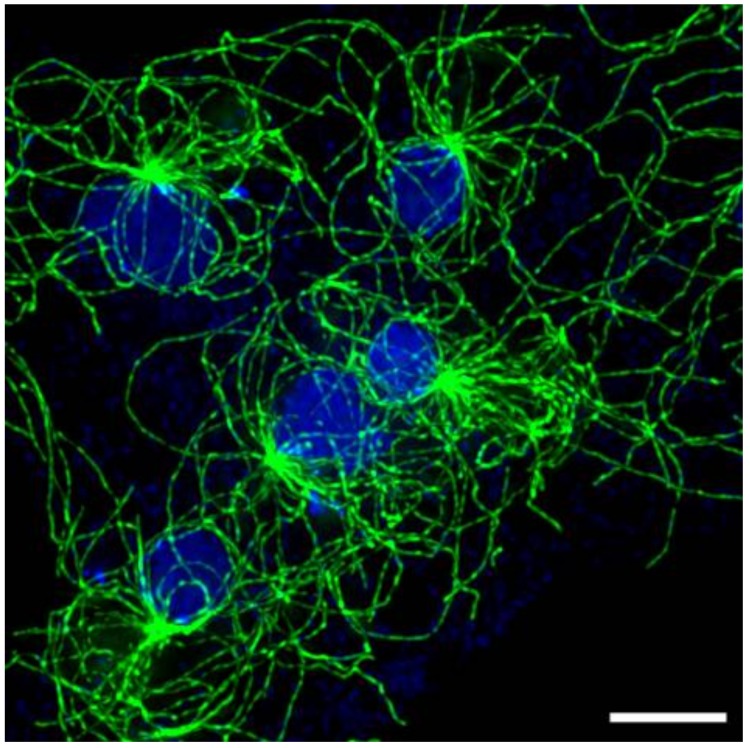
Multinucleated cell. Selected area of an exceptionally large, flattened cell containing multiple nuclei—five are visible here. Each nucleus is bound to a centrosome; each with a distinguishable MT array. Scale bar = 5 μm.

These observations indicate that multiple pushing and pulling forces acting on individual MTs play a dominant role in the architecture of the interphase MT array in *D. discoideum*. This mechanism is different than self-centering through MT assembly/disassembly and may be driven in large part by the biology of the *Dictyostelium* system. First, the amoebas are highly mobile; centrosome movement during cell crawling can occur at rates faster than repositioning through MT assembly/disassembly [16] and thus require an alternate mechanism to maintain cell polarity. Moreover, a striking feature of *D. discoideum* is its ability to accommodate multiple degrees of multinucleation. It is not uncommon for wild type cells to fail cytokinesis and generate multiple nuclei in the same cytoplasm (Figure 3). Targeted disruptions of myosin II have further generated exceptionally large syncytia with many nuclei [21,22,23]. These cells persist in culture, nuclei undergo synchronous division, and centrosomes can trigger multiple cleavage furrows that can reset cells to the mononucleated state.

In multinucleated syncytia, nuclei are generally spread uniformly throughout the cytoplasm and the multiple centrosome/MT arrays appear spatially segregated (Figure 3). And herein lies an interesting conundrum. In binucleates and greater, what forces are in play that sense and maintain individual MT arrays?

**Figure 4:**
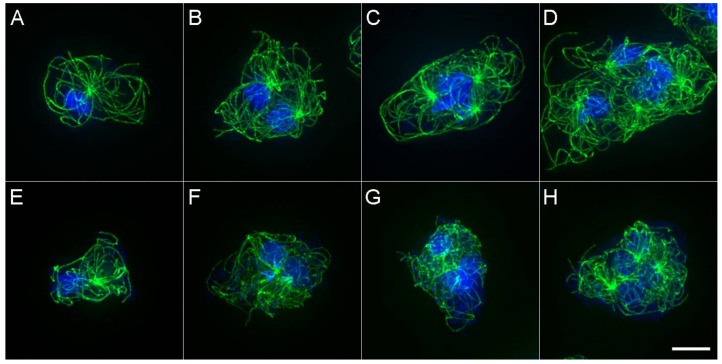
MT arrays in wild type and Ase1A null cells. Panels (**A**–**D**) show wild-type MT arrays in mono-, bi-, tri- and tetra-nucleated cells. Panels (**E**–**H**) show similar views of Ase1A null cells. Note in panels (**F**–**H**), the centrosomes are closer together and MT patterns are less distinct. Scale bar = 5 μm.

If MT arrays were uniformly subject to forces acting at MT tips, two arrays in a binucleate cell should converge into similar position and morphology, with centrosomes moving to the cell center. In vertebrate tissue culture cells, such centrosome clustering is a well-described adaptive process that minimizes the impact of extra centrosomes on mitotic fidelity [24]. Instead, the multiple arrays in a common *D. discoideum* cytoplasm seem positioned in ways that minimize interaction with one another. This observation suggests a mechanism that senses and reduces interaction between MT arrays, beyond that provided by simple motor protein and tip dynamics. To address this possibility, we targeted a MT effector protein family known to interact with MTs of opposite polarity (MAP65/Ase1/PRC1) [9]. In many animal cells, PRC1/Ase1 functions to organize MT overlap in the central spindle region during division and as anticipated, DdAse1A performs a similar mitotic function in *D. discoideum*. In addition, in plants and some fungi, MAP65/Ase1 isoforms also function during interphase to bundle MTs or facilitate nuclear spacing [25,26,27,28]. In *D. discoideum*, Ase1A removal decreases the spacing between centrosomes during interphase and muddles the distinction between MT arrays in a common cytoplasm (Figure 4).

These results suggest that a structural MAP performs a key sensing function, one that works in combination with multiple motor proteins (e.g., dynein, DdKif8, DdKif10) that push or pull MTs to organize interphase arrays in *Dictyostelium* sp. Testing this idea and understanding the relative contributions of each component is an active part of our current work.

## 3. Centrosome–Nuclear Attachment

During interphase, centrosomes in *D. discoideum* are located in the cytoplasm but are visibly and firmly attached to nuclei (e.g., Figure 3 in Ref. [18]). Two connection mechanisms have been described, one through a series of short electron dense fibers between the corona and the nuclear envelope [6] and the other involves a dynamic MT-based component [29]. The fibrous component is likely crucial for the physical tethering of the centrosome to the nuclear envelope and facilitates nuclear integration during mitosis. The MT-mediated component is probably most useful in keeping the two organelles near each other and reeling them back together if separated. There are important reasons to keep the two organelles in close proximity [30]. The most obvious is to have the centrosome available for nuclear envelope import at the onset of mitosis; a second reason is highlighted in multinucleated cells, to ensure single centrosome-nucleus pairing and prevent aberrant spindle formation. A third reason is to couple nuclei to force-generating machinery to maintain polarity as cells crawl about.

A few studies have generated supernumerary centrosomes that persist in the cytosol away from nuclei. This can occur either through disruption of centrosome–nuclear interactions or by triggering de novo formation pathways. These studies include overexpression of core centrosome components such as DdCP224 [31], mutations in dynein associated proteins such as Lis1 and DIC [19,32] and through deletion of a central motor domain kinesin, Kif9 [29]. The latter study illustrates a novel adaption of a Kin-I kinesin to anchor itself at the nuclear envelope and maintain a tight opposition between the centrosome and nucleus through depolymerization of the intervening MTs. Dynein provides a related function to connect nuclei and centrosomes via force-generating machinery acting on MTs in many metazoan systems; while we cannot exclude a similar role in *D. discoideum*, dynein alone is insufficient to maintain a robust attachment in the absence of Kif9.

**Figure 5:**
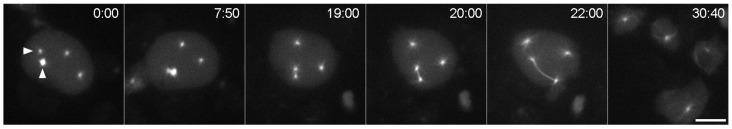
Spindle formation in presence of multiple centrosomes. Live cell sequence shows a mononucleated cell that contained two centrosomes prior to entering mitosis. In the first panel (time = 0:00, m:s), both centrosomes have duplicated and separated into four daughters, but only one daughter of the pair indicated with arrowheads contacts the nucleus (distinguished here because of the increase in GFP-tubulin fluorescence). By 7:50, the other daughter binds to the nucleus and the two centrosomes then cooperate to form a bipolar spindle. In this cell, all four centrosomes triggered cytokinetic furrows, cleaving the cell into two normal cells with nuclei and two cytoplasts (30:40). Scale bar = 5 μm.

There does not seem to be a significant penalty for not maintaining a tight centrosome–nuclear coupling during interphase. Cell crawling and response to chemotactic signals during development appear normal. However, defects are obvious as these cells enter mitosis. Centrosomes positioned in the cytoplasm away from the nucleus duplicate and separate, but do not form spindle like structures (Figure 5). Mitotic progression is stalled in cells where centrosomes fail to engage a nucleus [30]. Only the nuclear environment appears capable of supporting spindle formation, and MT assembly in the nucleus requires docking of at least one centrosome. If a single daughter centrosome contacts a nucleus, a monopolar spindle will form; two daughters attaching with similar timing will combine activity and build a bipolar spindle, more than two centrosomes will trigger formation of multipolar structures, all of which undergo some type of spindle elongation, astral MT extension during anaphase and extrusion back into the cytoplasm.

**Figure 6:**
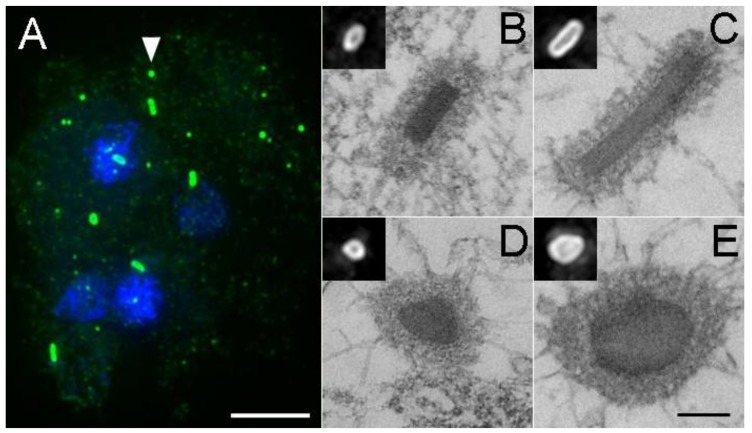
Aberrant centrosome formation. Panel (**A**) shows a tetranucleated cell with supernumerary centrosomes (green, DdCP224 staining, DdKif9 null background). The arrowhead highlights the normal appearance for an interphase centrosome. Note here the number, distribution, and variability in centrosome sizes, as well as the lack of nuclear association. Scale bar = 5 μm. Panels (**B**–**E**) show thin section electron micrographs of selected centrosomes. Scale bar = 250 nm (**B**,**D**) present side and top views of wild type centrosomes, (**C**,**E**) show elongated versions. Insets show the same centrosomes by light microscopy. Panels (**B**,**D**,**E**) adapted with permission from Ref [29].

If no type of centrosome–nuclear engagement occurs, cytoplasmic daughter centrosomes either undergo a reduplication cycle to generate numerous fragments of MT-nucleating structures, or after delay, cells simply exit mitosis and restore interphase MT arrays [30]. Both scenarios generate supernumerary centrosomes, some of which show size variations by light and electron microscopy (Figure 6) but remain competent to nucleate MTs in the subsequent interphase.

As illustrated in figure five (and Ref [31]), unattached centrosomes generate MT asters that remain competent to trigger cleavage furrows at the end of mitosis. In some cases, the furrows result in cytoplasts containing only the isolated MT array. In others, resulting cells accumulate additional centrosomes, each of which maintains an ability to spatially segregate from one another (Figure 6). These observations highlight the intrinsic capacity of the MT aster to segregate from one another, with minimal overlap. Cleavage furrow formation in the absence of chromatin has been addressed in vertebrate cell lines [33] and more recently in *Xenopus* sp. egg extracts [34,35], where asters minimize interdigitation of opposite polarity MTs and recruit the necessary components to partition cells. In parallel to *D. discoideum*, the Ase1 isoform (PRC1) and a kinesin homolog play significant roles in maintaining aster-aster segregation in *Xenopus* sp. [34,35]. Ultimately, the additional centrosomes increase the probability of multiple nuclear engagements and aberrant spindle formation that is unsustainable for cell viability.

From these observations, it is evident that centrosome–nuclear engagement is not required for centrosome duplication or daughter separation; centrosomes do not have a single, defined binding site on the nuclear envelope for mitotic integration; the nucleus is promiscuous and will engage multiple centrosomes if available; and in addition, it appears that the nucleus sequesters components that drive spindle formation.

The biology of the *Dictyostelium* sp. system highlights alternate strategies to position centrosomes and manage the radial character of MT arrays during interphase. Motors and associated proteins play dominant roles in MT organization and a kinesin isoform appears to have been adapted to supplant the dynein mechanism found in many metazoan systems to maintain centrosome-nuclear proximity. Some of the interesting questions this system is poised to address are the nature of the biochemical signals that trigger dissolution of the interphase MT array, what triggers nuclear import of mitotic components and what stimulates centrosome docking into the nuclear envelope. Although cyclins and mitotic checkpoint proteins have been identified in *D. discoideum* [36] and at least cyclin B functions as expected from other organisms [37], little is known about the machinery here that drives the centrosome transitions from interphase into mitosis and regulates the duplication cycle. Moreover, the longitudinal splitting of the centrosome during duplication and the maturation of the newly exposed surfaces to nucleate spindle MTs are striking events and offer a fresh approach to understanding how MT nucleators are recruited to centrosome binding sites and are activated.

## References

[1] Azimzadeh J. (2014). Exploring the evolutionary history of centrosomes. Philos. Trans. R. Soc. B Biol. Sci..

[2] Bornens M., Azimzadeh J. (2007). Origin and evolution of the centrosome. Eukaryotic Membranes and Cytoskeleton: Origins and Evolution.

[3] Carvalho-Santos Z., Azimzadeh J., Pereira-Leal J.B., Bettencourt-Dias M. (2011). Tracing the origins of centrioles, cilia and flagella. J. Cell Biol..

[4] Gräf R., Batsios P., Meyer I. (2015). Evolution of centrosomes and the nuclear lamina: Amoebozoan assets. Eur. J. Cell Biol..

[5] Bestul A.J., Yu Z., Unruh J.R., Jaspersen S.L. (2017). Molecular model of fission yeast centrosome assembly determined by superresolution imaging. J. Cell Biol..

[6] Omura F., Fukui Y. (1985). *Dictyostelium* MTOC: Structure and linkage to the nucleus. Protoplasma.

[7] Ueda M., Schliwa M., Euteneuer U. (1999). Unusual centrosome cycle in *Dictyostelium*: Correlation of dynamic behavior and structural changes. Mol. Biol. Cell.

[8] Tikhonenko I., Nag D.K., Martin N., Koonce M.P. (2008). Kinesin-5 is not essential for mitotic spindle elongation in *Dictyostelium*. Cell Motil. Cytoskelet..

[9] Tikhonenko I., Irizarry K., Khodjakov A., Koonce M.P. (2016). Organization of microtubule assemblies in *Dictyostelium* synctia depends on the microtubule crosslinker, Ase1. Cell. Mol. Life Sci..

[10] Holy T.E., Dogterom M., Yurke B., Leibler S. (1997). Assembly and positioning of microtubule asters in microfabricated chambers. Proc. Natl. Acad. Sci. USA.

[11] Malikov V., Cytrynbaum E.N., Kashina A., Mogilner A., Rodionov V. (2005). Centering of a radial microtubule array by translocation along microtubules spontaneously nucleated in the cytoplasm. Nat. Cell Biol..

[12] McNally F.J. (2013). Mechanisms of spindle positioning. J. Cell Biol..

[13] Dawe H.R., Farr H., Gull K. (2007). Centriole/basal body morphogenesis and migration during ciliogenesis in animal cells. J. Cell Sci..

[14] Pitaval A., Senger F., Letort G., Gidrol X., Guyon L., Sillibourne J., Théry M. (2017). Microtubule stabilization drives 3D centrosome migration to initiate primary ciliogenesis. J. Cell Biol..

[15] Gundersen G.G., Worman H.J. (2013). Nuclear positioning. Cell.

[16] Brito D.A., Strauss J., Magidson V., Tikhonenko I., Khodjakov A., Koonce M.P. (2005). Pushing forces drive the comet-like motility of microtubule arrays in *Dictyostelium*. Mol. Biol. Cell.

[17] Koonce M.P., Khodjakov A. (2002). Dynamic microtubules in *Dictyostelium*. J. Muscle Res. Cell Motil..

[18] Koonce M.P., Kohler J., Neujahr R., Schwartz J.M., Tikhonenko I., Gerisch G. (1999). Dynein motor regulation stabilizes interphase microtubule arrays and determines centrosome position. EMBO J..

[19] Rehberg M., Kleylein-Sohn J., Faix J., Ho T.H., Schulz I., Gräf R. (2005). *Dictyostelium* Lis1 is a centrosomal protein required for microtubule/cell cortex interactions, nucleus/centrosome linkage and actin dynamics. Mol. Biol. Cell.

[20] Nag D.K., Tikhonenko I., Soga I., Koonce M.P. (2008). Disruption of four kinesin genes in *Dictyostelium*. BMC Cell Biol..

[21] De Lozanne A., Spudich J.A. (1987). Disruption of the *Dictyostelium* myosin heavy chain gene by homologous recombination. Science.

[22] Knecht D.A., Loomis W.F. (1987). Antisense RNA inactivation of myosin heavy chain gene expression in *Dictyostelium* discoideum. Science.

[23] Neujahr R., Albrecht R., Kohler J., Matzner M., Schwartz J.M., Westphal M., Gerisch G. (1998). Microtubule-mediated centrosome motility and the positioning of cleavage furrows in multinucleate myosin II-null cells. J. Cell Sci..

[24] Quintyne N.J., Reing J.E., Hoffelder D.R., Gollin S.M., Saunders W.S. (2005). Spindle multipolarity is prevented by centrosomal clustering. Science.

[25] Anderson C.A., Eser U., Korndorf T., Borsuk M.E., Skotheim J.M., Gladfelter A.S. (2013). Nuclear repulsion enables division autonomy in a single cytoplasm. Curr. Biol..

[26] Ho C.-M.K., Hotta T., Guo F., Roberson R.W., Lee Y.-R.J., Liu B. (2011). Interaction of antiparallel microtubules in the phragmoplast is mediated by the microtubule-associated protein MAP65-3 in *Arabidopsis*. Plant Cell.

[27] Loiodice I., Staub J., Setty T.G., Nguyen N.P., Paoletti A., Tran P.T. (2005). Ase1p organizes antiparallel microtubule arrays during interphase and mitosis in fission yeast. Mol. Biol. Cell.

[28] Lucas J.R., Courtney S., Hassfurder M., Dhingra S., Bryant A., Shaw S.L. (2011). Microtubule-associated proteins MAP65-1 and MAP65-2 positively regulate axial cell growth in etiolated *Arabidopsis* hypocotyls. Plant Cell.

[29] Tikhonenko I., Magidson V., Gräf R., Khodjakov A., Koonce M.P. (2013). A kinesin-mediated mechanism that couples centrosomes to nuclei. Cell. Mol. Life Sci..

[30] Leo M., Santino D., Tikhonenko I., Magidson V., Khodjakov A., Koonce M.P. (2012). Rules of engagement: Centrosome-nuclear connections in a closed mitotic system. Biol. Open.

[31] Gräf R., Euteneuer U., Ho T.-H., Rehberg M. (2003). Regulated expression of the centrosomal protein DdCP224 affects microtubule dynamics and reveals mechanisms for the control of supernumerary centrosome number. Mol. Biol. Cell.

[32] Ma S., Triviños-Lagos L., Gräf R., Chisholm R.L. (1999). Dynein intermediate chain mediated dynein–dynactin interaction is required for interphase microtubule organization and centrosome replication and separation in *Dictyostelium*. J. Cell Biol..

[33] Savoian M.S., Earnshaw W.C., Khodjakov A., Rieder C.L. (1999). Cleavage furrows formed between centrosomes lacking an intervening spindle and chromosomes contain microtubule bundles, INCENP and CHO1 but not CENP-E. Mol. Biol. Cell.

[34] Nguyen P.A., Field C.M., Mitchison T.J., Surrey T. (2018). Prc1E and Kif4A control microtubule organization within and between large *Xenopus* egg asters. Mol. Biol. Cell.

[35] Nguyen P.A., Groen A.C., Loose M., Ishihara K., Wühr M., Field C.M., Mitchison T.J. (2014). Spatial organization of cytokinesis signaling reconstituted in a cell-free system. Science.

[36] Huber R.J. (2014). The cyclin-dependent kinase family in the social amoebozoan *Dictyostelium discoideum*. Cell. Mol. Life Sci..

[37] Luo Q., Michaelis C., Weeks G. (1994). Overexpression of a truncated Cyclin B gene arrests *Dictyostelium* cell division during mitosis. J. Cell Sci..

[38] Basu S., Fey P., Pandit Y., Dodson R., Kibbe W.A., Chisholm R.L. (2013). Dictybase 2013: Integrating multiple Dictyostelid species. Nucleic Acids Res..

